# Netflix and Chill? What Sex Differences Can Tell Us About Mate Preferences in (Hypothetical) Booty-Call Relationships

**DOI:** 10.1177/1474704918812138

**Published:** 2018-11-14

**Authors:** Evita March, George Van Doorn, Rachel Grieve

**Affiliations:** 1 School of Health and Life Sciences, Federation University Australia, Victoria, Australia; 2 University of Tasmania, Hobart, Tasmania, Australia

**Keywords:** mate preferences, booty calls, short-term relationship, long-term relationship, mating, mate budget

## Abstract

The booty-call relationship is defined by both sexual characteristics and emotional involvement. In the current study, men’s and women’s preferences for a booty-call mate were explored. Men and women were predicted to exhibit different mate preferences depending on whether they considered a booty-call relationship a short- or long-term relationship. Participants (*N* = 559, 74% women) completed an anonymous online questionnaire, designing their ideal booty-call mate using the mate dollars paradigm. Both sexes considered the physical attractiveness and kindness of a booty-call mate a necessity, expressing both short- and long-term mate preferences. The current study highlights the need to explore mate preferences outside the dichotomy of short- and long-term relationships, providing evidence of a compromise relationship.

Sex differences in mate preferences are predominantly considered in the context of long-term, committed relationships (e.g., Buss, 1989; Li, Bailey, Kenrick, & Linsenmeier, 2002) and casual, short-term sexual relationships (e.g., Li & Kenrick, 2006). However, recent research has noted that not all romantic relationships fall into the dichotomy of short- or long term (Jonason, 2013; Jonason, Valentine, & Li, 2012; March & Grieve, 2015; Wentland & Reissing, 2011). The spectrum of relationships individuals engage in includes booty calls (Jonason, Li, & Cason, 2009; Jonason, Li, & Richardson, 2011), fuck buddies (Wentland & Reissing, 2011), and friends with benefits (Bisson & Levine, 2009), among others. If these relationships are legitimate in their own right (i.e., they exist outside the dichotomy of short- and long-term relationships), there is a paucity of research concerning mate preferences within each relationship paradigm. The aim of this study was to consider, for the first time, the characteristics men and women consider necessities in a potential booty-call mate; a liaison that has elements of both short- and long-term relationships. In addition, exploring the characteristics men and women consider necessities in a booty-call partner will shed light on whether men and women consider the booty call a short-term, unemotional interaction, or a short-term interaction that has the potential to develop into a long-term relationship.

## Long-Term Mate Preferences

In regard to long-term, potential mates, men rank the physical attractiveness of a mate as being more important than do women, while women rate the status and resources of a mate as more important than do men (e.g., Hill & Reeve, 2004; March & Bramwell, 2012; March & Grieve, 2014; Shackelford, Schmitt, & Buss, 2005). These sex differences are found to be reliable and consistent across cultures (Buss, 1989; Buss et al., 1990). In addition, studies have found the trait of kindness is valued equally by the sexes (e.g., Buss, 1989), with both men and women considering a long-term mate’s kindness a necessity (Li et al., 2002). However, some studies have shown that women place a higher priority on a mate’s kindness than do men (Evans & Brase, 2007). Both evolutionary and social–economic theory attempt to elucidate the origins of these preferences in a long-term mate.

As modern dating behavior is considered to reflect evolved adaptations (Stanik & Ellsworth, 2010), these mate preferences have been attributed to evolutionary mechanisms. According to evolutionary theory, as the reproductive costs are higher for women (e.g., internal gestation, extended parental care; Trivers, 1972), women have come to value a long-term mate who has the ability to contribute the resources necessary to ensure the survival of any resulting offspring (Buss, 2006; Buunk, Dijkstra, Fetchenhauer, & Kenrick, 2002). Meanwhile, as men’s reproductive success is constrained by access to fertile women (Tadinac, 2010), men have come to value qualities (e.g., physical attractiveness) that reflect reproductive potential in budding mates (Montoya, 2005). Women may also seek a mate who is kind, as kindness may indicate that their potential mate is willing to share their resources (Jensen-Campbell, Graziano, & West, 1995) and be a better, more attentive parent (Urbaniak & Kilmann, 2006). Thus, evolutionary theory adequately explains why women consider a mate’s kindness more important than do men (e.g., Evans & Brase, 2007) and why kindness is important for both men and women when selecting long-term partners (i.e., is likely a cue to good nurturing ability; see Buss, 1989). Both evolutionary theory and social–economic theory highlight the importance of adjusting to the environment (Eagly & Wood, 1999) and are not considered inherently incompatible (Buss & Barnes, 1986; Feingold, 1990; Howard, Blumstein, & Schwartz, 1987). However, evolutionary research has been criticized for a heavy focus on between-sex differences in mate preferences rather than within-sex differences in mate preferences (Gangestad & Simpson, 2000; Walter, 1997).

To address these within-sex differences, social theories attribute sex differences in mate preferences to social roles adopted by men and women (social role theory; Eagly & Wood, 1999) and economic constraints the sexes face (Moore & Cassidy, 2007). Social role theory proposes that historical labor divisions have led men and women to take on different social roles, with this occupation of different roles resulting in development of gender roles (Eagly & Steffen, 1984). Traditionally, men secure higher paying jobs and higher status professions relative to women (Hamida, Mineka, & Bailey, 1998). Consequently, women’s ability to provide for themselves has been historically constrained (Moore & Cassidy, 2007). Because of the restrictions women face regarding individual advancement, women seek in mates the characteristics that have historically been denied to them (i.e., status and resources; Buss & Barnes, 1986). As men have not experienced the same historical economic constraints, men are able to focus their initial search on the physical attractiveness of a mate.

## Short-Term Mate Preferences

Both sexes pursue and engage in short-term, sexual relationships (see Strout, Fisher, Kruger, & Steeleworthy, 2010). As such, researchers have contrasted the preferences people show for a short-term mate (e.g., one-night stand) with preferences for a long-term mate (e.g., spouse; Scheib, 2001). With regard to short-term mates, both men and women have been found to place the most emphasis on the mate’s physical attractiveness (Wiederman & Dubois, 1998). For example, Buunk, Dijkstra, Fetchenhauer, and Kenrick (2002) showed that both sexes desire a higher level of physical attractiveness as relationship lengths shorten.

Given this information, it seems that little changes for men across relationship types (i.e., physical attractiveness is prioritized), but that the story is more interesting for women. Unmistakable in the existing research is that women prefer physically attractive mates for short-term relationships and mates with high status and resources for long-term relationships (Hill & Reeve, 2004; March & Bramwell, 2012; Schulte-Hostedde, Eys, & Johnson, 2008; Shackelford et al., 2005). It is perhaps the case that women adapt their mating strategies as a consequence of the nature of short-term relationships, thus prioritizing a mate’s genetic quality over status and resources. Strategic pluralism theory (Gangestad & Simpson, 2000) posits that individuals will engage in different mating strategies according to environmental conditions and relationship styles. By recognizing individual differences in mating strategies and environments, strategic pluralism theory can adequately account for the diversity of women engaging in short-term mating. For example, individuals use serious romantic relationships to gain socioemotional support and one-night stands to gain sexual gratification (Jonason, 2013).

Alternatively, sexual strategies theory (Buss & Schmitt, 1993) suggests that women may use short-term mating as a means to evaluate mates as potential long-term partners (see Jonason et al., 2009). Women might use short-term sexual relationships to identify and acquire a long-term partner by gauging the benefits gained when in the short-term relationship (Greiling & Buss, 2000). Taken together, both sexual strategies theory and strategic pluralism theory can account for mating strategies of men and women. However, although some women may engage in short-term relationships as a means to identify potential long-term mates (i.e., sexual strategies theory), women may still engage in short-term relationships for reasons other than acquiring a long-term mate, such as securing good genes that will benefit potential offspring (Kruger, Fisher, & Jobling, 2003; Vigil, Geary, & Byrd-Craven, 2006).

## The Nature of Booty-Call Relationships

Research on sex differences in mate preferences has predominantly focused on two “polar-opposite relationship types” (Jonason, Li, & Richardson, 2011, p. 486): short term and long term (see also Aitken, Lyons, & Jonason, 2013; Jonason et al., 2009). However, not all human relations fall precisely within these two categories. Some relationships incorporate elements of both short- and long-term relationships, an example being the booty call. The booty call is characterized by a relationship that is not committed or expected to be monogamous (Singer et al., 2006) but incorporates repeated sexual encounters (Jonason et al., 2012). By definition, a booty call involves contacting a non-long-term mate with the primary purpose of engaging in sexual activity. This contact is most commonly made via telephone (Jonason et al., 2009) or by text message (Wentland & Reissing, 2011). Spontaneous contact is considered to be a key feature of the booty-call relationship.

The booty-call relationship has been conceptualized as a “compromise” relationship between the sexes (Jonason et al., 2009 , 2011). According to this premise, it consists of sexual encounters with lower investment than a committed relationship (and is thus appealing to men) but has an element of commitment greater than that of a one-time sexual encounter (and is thus appealing to women). Wentland and Reissing (2011) reported that individuals engaged in a booty call do not consider the other party a friend (and, as such, differs from the friends with benefits relationship) and thus do not socialize with one another (see also Jonason et al., 2011). Further, Wentland and Reissing (2011) reported that the booty call does not involve emotional investment and is characterized by an “unemotional, perfunctory manner” (p. 87). However, Jonason and colleagues (2011) showed that, although the booty-call relationship often lacks the emotional acts found in serious, long-term relationships (such as talking and handholding), more emotional, intimate acts were found to occur more often in booty-call relationships relative to one-night stands. For example, kissing, manual sex, fondling of breasts/chest, and anal sex were reported to occur significantly more often in booty calls than in one-night stands.

As is evident above, there are differences in the defining qualities of a booty call. On the one hand, the booty-call relationship is characterized as unemotional and exists purely for spontaneous, sexual gain (Jonason, 2013). This definition is supported by findings showing that both men and women accept or reject a booty call based on the initiator’s physical attractiveness (Jonason et al., 2009). On the other hand, the booty-call relationship may involve more emotional involvement and time than a short-term, casual sex relationship and thus gives women the opportunity to screen the booty-call participant as a potential long-term mate (Jonason et al., 2011). This idea is supported by findings showing that men were more likely than women to report that a booty call did not transition into a long-term relationship as the men were only interested in a sexual relationship (Jonason et al., 2009). Women, on the other hand, were more likely to report that the booty call did not transition into a long-term relationship because the other person was not interested in a long-term relationship. Jonason, Li, and Cason (2009) argue that this result is substantial support for the claim that men tend to view booty calls as mostly sexual, whereas women may have some level of emotional involvement.

## Aim and Hypotheses

The current study aimed to assess the characteristics considered necessities in a booty-call mate, an area which has not yet received attention in the literature. This will help elucidate whether men and women consider the booty-call relationship purely short term or a short-term relationship with long-term potential. The current study will build on previous research of Li, Bailey, Kenrick, and Linsenmeier (2002) and Li and Kenrick (2006).

Previous research has shown men consider physical attractiveness a necessity in both long- and short-term mates (Li et al., 2002; Li & Kenrick, 2006). Women, however, consider social level (i.e., status and resources) and kindness a necessity in a long-term mate and physical attractiveness a necessity in a short-term mate. Li and colleagues (2002) define a necessity as a characteristic that is initially sought in a mate, and after this characteristic is obtained, the search for other characteristics (defined as luxuries) begins. Here, a necessity is defined as a mate characteristic that *must* be satisfied in order to engage in a booty call; once a necessity is satisfied, other desirable characteristics can be sought (described as luxuries; Li et al., 2002).

Studying the characteristics men and women consider necessities in a booty-call mate should reveal (1) whether women consider a booty-call mate a potential long-term partner, (2) whether the physical attractiveness of a potential booty-call mate is actually a necessity, and (3) if kindness is a necessity for men and women in a booty-call mate (kindness is a characteristic not commonly valued in short-term mates but is considered by both sexes as highly desirable in long-term mates; Buss & Barnes, 1986). To properly assess the mate preferences for a booty-call relationship, mate preferences regarding long- and short-term mates were also assessed. On the basis of previous research, if both men and women consider a booty call a short-term, unemotional relationship (e.g., Wentland & Reissing, 2011), then:

**Hypothesis 1:** Both men and women will consider physical attractiveness a necessity.
**Hypothesis 2:** Both men and women will consider kindness a luxury.
**Hypothesis 3:** Women will consider social level a luxury.

However, if a booty-call relationship is a hybrid relationship that helps reach a compromise between the sexes—offering men sexual encounters with limited (although some) emotional investment and women with sexual encounters alongside the opportunity to trial run a potential long-term mate (e.g., Jonason et al., 2009 , 2011)—then:

**Hypothesis 4:** Both men and women will consider physical attractiveness a necessity.
**Hypothesis 5:** Both men and women will consider kindness a necessity.
**Hypothesis 6:** Women will consider social level a necessity.

## Method

### Participants

There were 559 participants with a mean age of 24.03 years (*SD* = 11.05), with 2 participants not supplying their age. Of the participants, 26.48% (148 people) were men, and 73.52% (411 people) were women. Regarding sexual orientation, 87.84% (491 people) were heterosexual, 6.44% (36 people) were homosexual, 5.19% (29 people) were bisexual, and 0.54% (3 people) identified as “Other.” For men, 54.76% had previously been involved in a booty-call relationship, whereas 58.14% of women had previously been involved in a booty-call relationship. For men, 9.52% were currently involved in a booty-call relationship, whereas 7.06% of women were currently involved in a booty-call relationship. Finally, 58% (324 people) were current university students. There were no selection criteria, other than being aged 18 years or older (i.e., participants were not required to be in a relationship). A power analysis (G*Power; Faul, Erdfelder, Lang, & Buchner, 2007) indicated that a sample size of 155 was required to yield power of 80% to detect a medium effect size of at least 0.25 (α = .05). The current sample size (*N* = 559) was therefore considered to have adequate power to yield reliable results.

### Materials

An anonymous online questionnaire included a demographics section and a mate budget. Demographics sought information about participant’s age, sex, current education status, if participants had ever been involved in a booty-call relationship, and if participants were currently involved in a booty-call relationship. The booty-call relationship was defined for participants as “an uncommitted relationship where communication (e.g., phone call, texting) only takes place when there is the urgent intent, either stated or implied, of having sexual activity and/or intercourse” (see Jonason et al., 2009).

The current study used the mate budget paradigm (e.g., Jonason, Luevano, & Adams, 2012; Li et al., 2002; March & Grieve, 2015). The mate budget paradigm requires participants to spend hypothetical mate dollars on five traits (physical attractiveness, creativity, kindness, liveliness, and social level) on a scale ranging from 0 to 100 percentiles. Before spending the mate dollars, participants are provided with a brief description of each characteristic and an overview of the budget allocation method. Although all characteristics are included in the budget, only physical attractiveness, kindness, and social level were analyzed.

There were two conditions: low budget (10 mate dollars) and high budget (30 mate dollars). Participants were asked to spend all their mate dollars on the five characteristics in each condition, and presentation of the low and high budget was counterbalanced (46.5% of participants received the low budget first). Characteristics that received the most mate dollars when the budget was low were considered mate traits men and women considered a necessity, and characteristics that received the most mate dollars when the budget was high were considered mate traits men and women considered a luxury.

To complete the mate budget, participants were randomly allocated into one of the three conditions: long term, short term, and booty call. For long term, participants were asked to spend mate dollars to design their ideal long-term mate (someone they might wish to marry). For short term, participants were asked to spend mate dollars to design their ideal short-term mate (someone they may have casual sex with for one evening). For booty call, participants were asked to spend mate dollars to design their ideal booty-call mate (someone with whom they will communicate with over a long-term period with the intent of short-term sexual gratification).

### Procedure

Participants were recruited on and off an Australian university campus, with the study promoted as investigating personality and relationships. Participants on campus were recruited via hard copy advertisements that informed participants of the voluntary, anonymous, and online questionnaire. The posted advertisement provided the web link to the online questionnaire. Off campus participants were recruited via social media advertisements that contained the same information as the hard copy advertisements. Participants were informed that the online questionnaire would take roughly 10 min of their time to complete. Upon completion of the questionnaire, participants were thanked and scores were amalgamated into the data file.

## Results

Three 3 × 2 × 2 mixed-models analyses of variance were conducted with type of relationship (short term, booty call, and long term) and gender (men and women) as the between-subjects independent variables, budget (low and high) as the within-subjects independent variable, and the three mate characteristics of physical attractiveness, kindness, and social level as the dependent variables (see Table 1 for descriptive statistics). Table 2 presents a summary of the main effects, and the full report of the main effect analyses can be found in Appendix.

**Table 1.** Mean Percentages Allocated to Each Characteristic for Men and Women in Low and High Budgets for Short-Term Mates, Booty-Call Mates, and Long-Term Mates.

Characteristics	Men		Women		Total	
	Low Budget	High Budget	Low Budget	High Budget	Low Budget	High Budget
	*M* (*SD*)	*M* (*SD*)	*M* (*SD*)	*M* (*SD*)	*M* (*SD*)	*M* (*SD*)
Short-term relationship						
Physical attractiveness	40.71 (15.62)	27.61 (4.90)	32.74 (11.66)	24.54 (5.12)	34.53 (13.05)	25.23 (5.22)
Kindness	17.22 (9.09)	17.93 (6.34)	21.85 (8.14)	23.06 (5.74)	20.80 (8.56)	21.90 (6.24)
Social level	13.97 (7.75)	17.83 (7.00)	16.75 (7.40)	18.69 (5.09)	16.12 (7.55)	18.49 (5.57)
Booty-call relationship						
Physical attractiveness	44.64 (19.99)	25.50 (5.99)	30.54 (18.84)	23.37 (5.72)	34.47 (20.82)	24.09 (5.87)
Kindness	19.51 (16.96)	20.29 (7.37)	29.06 (15.98)	23.62 (6.84)	25.85 (16.85)	22.50 (7.17)
Social level	10.61 (10.76)	17.88 (6.34)	12.65 (9.19)	18.77 (6.43)	11.97 (9.75)	18.47 (6.39)
Long-term relationship						
Physical attractiveness	25.87 (14.22)	21.13 (6.65)	20.61 (7.61)	20.14 (3.90)	21.69 (9.54)	20.35 (4.59)
Kindness	26.41 (6.76)	23.69 (5.03)	30.35 (8.38)	25.45 (4.08)	29.54 (8.22)	25.09 (4.34)
Social level	15.98 (10.22)	18.96 (5.59)	17.91 (9.30)	19.39 (5.76)	17.51 (9.50)	19.30 (5.71)

**Table 2.** Summary of Main Effects for Gender, Relationship Type, and Budget on Characteristics of Physical Attractiveness, Kindness, and Social Level.

Characteristics	*F* test	$\eta_{\text{p}}^{2}$
Physical attractiveness		
Sex	*F*(1, 493) = 40.46***	.08
Relationship type	*F*(2, 493) = 53.45***	.18
Budget	*F*(1, 493) = 208.33***	.30
Kindness		
Sex	*F*(1, 493) = 38.37***	.07
Relationship type	*F*(2, 493) = 27.43***	.10
Budget	*F*(1, 493) = 13.56***	.03
Social level		
Sex	*F*(1, 493) = 4.69*	.01
Relationship type	*F*(2, 493) = 6.24**	.03
Budget	*F*(1, 493) = 93.16***	.16

*p < .05. **p < .01. ***p < .001.

### Physical Attractiveness

For physical attractiveness, there was a significant two-way interaction between budget and gender, *F*(2, 493) = 25.10, *p* = .001, $\eta_{\text{p}}^{2}$ = .05. In addition, there was a significant two-way interaction between budget and relationship type, *F*(2, 493) = 22.85, *p* = .001, $\eta_{\text{p}}^{2}$ = .09. No other interactions reached significance.

**Figure 1.**
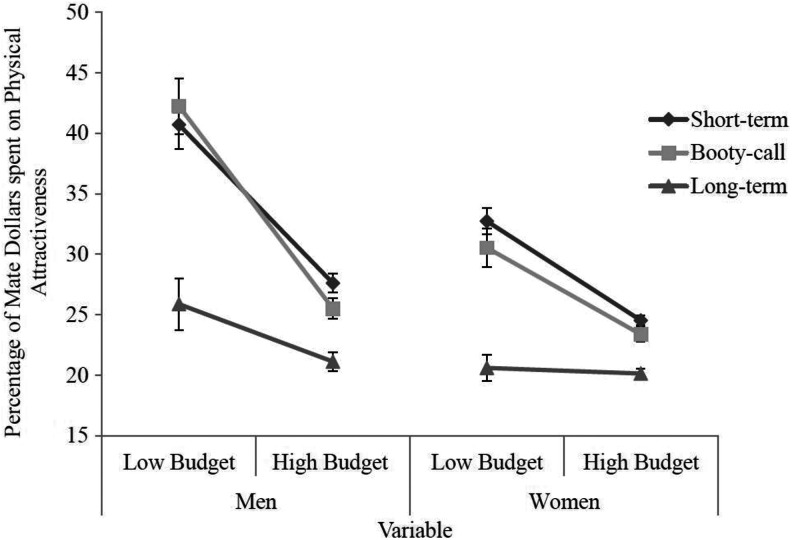
Three-way interaction for variables of budget, gender, and type of relationship for percentage of mate dollars spent on physical attractiveness. Error bars represent standard error. *Y*-axis begins at 15%.

Post hoc tests with a Bonferroni correction showed that both men and women spent more mate dollars on physical attractiveness in the low budget condition than they did in the high budget condition, *p* = .001, and for each type of relationship (short term, booty call, and long term), both men and women spent significantly more mate dollars in the low budget than the high budget, *p* = .001, .001, and .011, respectively. Although the omnibus three-way interaction did not reach statistical significance, post hoc tests revealed significant results. However, due to the nonsignificance of the overall test, these results should be interpreted with caution. For short-term relationships, both men (*p* = .001) and women (*p* = .001) spent significantly more mate dollars on physical attractiveness in the low budget compared to the high budget condition. In addition, for booty-call relationships, both men (*p* = .001) and women (*p* = .001) spent significantly more mate dollars on physical attractiveness in the low budget compared to the high budget condition. However, for long-term relationships, only men (*p* = .001) and not women (*p* = .612) spent significantly more mate dollars on physical attractiveness in the low budget compared to the high budget condition. This three-way interaction is visually depicted in Figure 1.

### Kindness

For kindness, there was a significant two-way interaction between budget and gender, *F*(1, 493) = 7.86, *p* = .005, $\eta_{\text{p}}^{2}$ = .02. Although the interaction between gender and relationship type did not reach significance, there was a significant two-way interaction between budget and relationship type, *F*(2, 493) = 10.29, *p* = .001, $\eta_{\text{p}}^{2}$ = .04. Finally, there was a significant three-way interaction for budget, gender, and relationship type, *F*(2, 493) = 4.04, *p* = .018, $\eta_{\text{p}}^{2}$ = .02.

In relation to the significant interaction between budget and gender, post hoc tests with a Bonferroni correction showed that women spent significantly more mate dollars on kindness in the low budget compared to the high budget, *p* = .001. In relation to the significant interaction between budget and relationship type, both men and women spent significantly more mate dollars in the low budget than the high budget condition, *p* = .011, and .001, respectively. No other comparisons reached significance.

**Figure 2.**
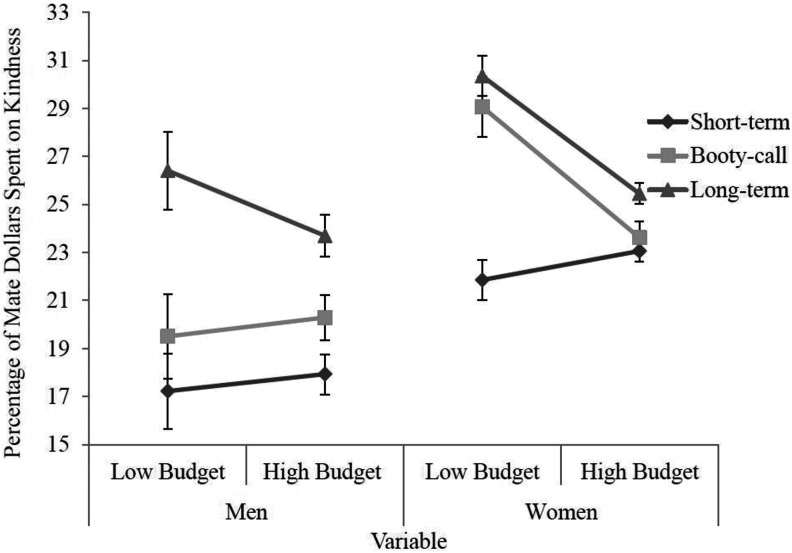
Three-way interaction for variables of budget, gender, and type of relationship for percentage of mate dollars spent on kindness. Error bars represent standard error. *Y*-axis begins at 15%.

For the significant three-way interaction of budget, gender, and relationship type, post hoc comparisons demonstrated that for booty-call relationships, only women spent significantly more mate dollars on kindness in the low budget compared to the high budget, *p* = .001. This result indicates that women, not men, consider the kindness of a booty-call mate a necessity. For short-term mates, there were no significant comparisons. Finally, for long-term relationships, both men (*p* = .047) and women (*p* = .001) spent significantly more mate dollars on kindness in the low budget compared to the high budget, suggesting that both men and women consider the kindness of a long-term mate a necessity. This three-way interaction is visually depicted in Figure 2.

### Social Level

For social level, there was no significant two-way interaction between budget and gender, *F*(1, 493) = 3.47, *p* = .063, $\eta_{\text{p}}^{2}$ = .01. There was, however, a significant two-way interaction between budget and relationship type, *F*(2, 493) = 10.32, *p* = .001, $\eta_{\text{p}}^{2}$ = .04. There was no significant two-way interaction between gender and relationship type, *F*(2, 493) = .08, *p* = .920, $\eta_{\text{p}}^{2}$ = .01. Finally, there was no significant three-way interaction for budget, gender, and relationship type, *F*(2, 493) = .07, *p* = .930, $\eta_{\text{p}}^{2}$ = .01.

To further explore these interactions, post hoc tests with a Bonferroni correction were conducted. For the interaction of budget and relationship type, post hoc tests showed that for all relationship types, individuals spent significantly more mate dollars in the high budget compared to the low budget, *p* = .001, .001, and.001 for short term, booty calls, and long term, respectively.

**Figure 3.**
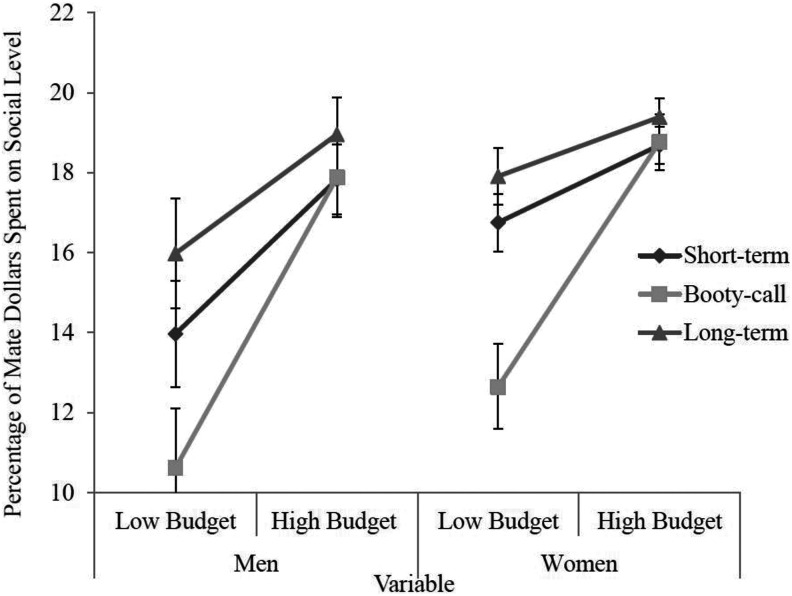
Three-way interaction for variables of budget, gender, and type of relationship for percentage of mate dollars spent on social level. Error bars represent standard error. *Y*-axis begins at 10%.

Although the omnibus three-way interaction did not reach statistical significance, post hoc tests revealed significant results. However, due to the nonsignificance of the overall test, these results should be interpreted with caution. Post hoc comparisons show that for short-term relationships, both men (*p* = .001) and women (*p* = .002) spent significantly more mate dollars on social level in the high budget compared to the low budget. In addition, for booty-call relationships, both men (*p* = .001) and women (*p* = .001) spent significantly more mate dollars on social level in the high budget compared to the low budget. Finally, for long-term relationships, both men (*p* = .013) and women (*p* = .015) spent significantly more mate dollars on social level in the high budget compared to the low budget. These results suggest that for these three relationship types, both men and women consider social level a luxury. This three-way interaction is visually depicted in Figure 3.

## Discussion

The aim of the current study was to assess the characteristics considered necessities in a booty-call mate, an interpersonal relationship which has received limited attention in the literature. Predictions were based on whether the booty-call relationship is considered a short-term, unemotional relationship or a hybrid long- and short-term relationship. To properly assess the mate preferences for a booty-call relationship, mate preferences regarding long- and short-term mates were also assessed.

### Long- and Short-Term Mate Preferences

For short-term mates, although the omnibus test for the three-way interaction did not reach significance, significant post hoc tests indicated that both men and women considered the physical attractiveness of a short-term mate necessity—a result further supported by the significant two-way interaction between budget and relationship type. The result of both sexes considering the physical attractiveness of a short-term mate a necessity corroborates the results of Li and Kenrick (2006). Furthermore, this result provides support for the premise that when considering a short-term mate, women place increased emphasis on physical attractiveness (Buunk et al., 2002; Wiederman & Dubois, 1998). The current results showed that both men and women did not consider the kindness or the social level of a short-term mate a necessity, in line with Li and Kenrick (2006). Interestingly, Li and Kenrick reported that men considered the kindness of a short-term mate a luxury—a result not replicated here. It is possible that as the mate budget has had limited use in the literature, the results are still relatively inconsistent.

Only men considered the physical attractiveness of a long-term mate a necessity, further corroborating previous research of Li and colleagues (2002). In addition, both men and women considered the kindness of a long-term mate a necessity and the social level of a long-term mate a luxury. Although Li and colleagues (2002) established that only women, not men, considered a long-term mate’s kindness as a necessity, both sexes have been shown to consider kindness as one of the most important and desirable traits for a potential romantic partner to possess (Buss & Barnes, 1986; Lippa, 2007). However, women considering the social level of a long-term mate a luxury, not a necessity, are inconsistent with the results of Li et al. (2002).

Although inconsistent, and as mentioned above, it should be noted that only a small body of research has used the mate budget paradigm. As such, characteristics men and women consider necessities and luxuries in mate preferences may not yet be established. It should be noted that the current results do not suggest that women do not care about the social level of a mate (nor do they suggest that men do not care about the social level of a mate) but simply may not consider this characteristic a necessity. Previous research posits many factors (e.g., gender roles, level of income) may influence a woman’s desire for a mate to possess significant status and resources (e.g., Eagly, Eastwick, & Johannesen-Schmit, 2009; Moore, Cassidy, & perrett, 2010). As such, women not considering a long-term mate’s social level a necessity may not be due to methodological limitations, but rather individual differences within and between samples. Importantly, this result provides support for strategic pluralism theory (Gangestad & Simpson, 2000), as women may be strategically adapting their mate preferences according to their environment.

### Booty-Call Mate Preferences

Based on previous studies (Jonason et al., 2009, 2011; Li et al., 2002; Li & Kenrick, 2006) regarding short-term, booty-call, and long-term relationships, we predicted that if both men and women consider a booty call a short-term, unemotional sexual relationship, physical attractiveness should be a necessity for both sexes, with kindness and social level as luxuries. Results were that, regardless of gender, physical attractiveness was considered a necessity in a booty-call mate. However, women were found to consider the kindness of a booty-call mate a necessity. As women did not consider the kindness of a short-term mate a necessity, these results do not support the premise that the booty-call relationship is considered a short-term, unemotional sexual relationship. Importantly, it should be noted that both men and women consider the kindness of a long-term mate a necessity. Combined with the current result that both men and women consider the physical attractiveness of a booty call, a mate, and a necessity, results of the current study support the premise of Jonason and colleagues (2011) who proposed the booty-call relationship as a sexual relationship but more emotional than the short-term, one-night stand relationship. Thus, results of the current study best support the second hypothesis, which proposed that a booty-call relationship may be a hybrid long- and short-term relationship that helps reach a compromise between the sexes.

However, it should be noted that the compromise relationship appears to only be the case for women, not men. Although women’s booty-call mate preferences appeared to be an amalgamation of short- and long-term mate preferences, men’s booty-call mate preferences mirrored their short-term mate preferences. Thus, the current study appears to support Jonason and colleagues’ (2009) suggestion that the booty call may be characterized as a “compromise relationship” between the sexes (see Jonason et al., 2009) in that it allows men to have sex without a high level of commitment, while offering women the *potential* for future commitment. Results of the current study also support the premise that men and women differ more in preferences when considering primarily sexual relationships (e.g., Jonason, 2013), further supporting sexual strategies theory (Buss & Schmitt, 1993). The results of the current study provide further conceptualization of new contemporary relationship styles in comparison to traditional styles (e.g., long-term marriage, short-term casual one-night stand).

Finally, although the omnibus test did not reach significance and thus results be interpreted with caution, post hoc comparisons showed both men and women considered the social level of a booty-call mate a luxury. Interestingly, social level was considered by both sexes to be a luxury across all types of relationships (short term, booty call, and long term). Given this consistency, it appears that men’s and women’s preference for a booty-call mate’s social level is reflective of their short- and long-term mate preferences. Thus, it can still be said that booty-call mate preferences are an amalgamation of both short- and long-term mate preferences.

### Limitations and Future Directions

A potential limitation of the current study was that the list of characteristics (i.e., physical attractiveness, kindness, and social level) was short. Although this list of traits was consistent with previous work in this area (e.g., Li et al., 2002), traits not assessed or explored here might be deemed important in a potential booty-call mate. Future research could assess additional mate characteristics, such as intelligence (Lippa, 2007), creativity (e.g., Li et al., 2002), and even other traits that may be considered more important in a primarily sexual relationship, such as eroticism and sexual performance.

A further limitation is the relatively small sample size. Although post hoc tests reached significance, overall omnibus tests did not. This, combined with the effect sizes of these tests, suggests that the power of the test may have been constrained by the sample size. Future research should seek to recruit a larger number of participants when conducting comparisons between relationship types. Nonetheless, the sample size for the current study (*N* = 559) was substantially larger than previous research examining booty-call relationships (e.g., *N* = 123 in Collins & Horn, 2018; *N* = 61 in Jonason et al., 2009; *N* = 123 in Jonason et al., 2011; *N* = 192 in Wesche, Claxton, Lefkowitz, & van Dulman, 2017), and because our sample included a substantial proportion of nonstudents, we suggest that our results provide reasonable insight into this particular interpersonal behavior.

The results of the current study may also be limited in generalizing to all sexual orientations, as the sample was predominantly heterosexual (88.2%). Although the mating strategies of homosexual and heterosexual men and women are not considered to differ (Symons, 1979), some research has shown differences in mate preferences for homosexual and heterosexual women (Bailey, Gaulin, Agyei, & Gladue, 1994). As such, although homosexual men’s and women’s mate preferences may be fundamentally similar to their heterosexual counterparts, this similarity should not be assumed (March, Grieve, & Marx, 2014). Future research would benefit from exploring mate preferences of individuals other than those with a heterosexual orientation in these relationship paradigms (i.e., booty calls, friends with benefits, and fuck buddies).

## Conclusion

An apparent flaw in much of the existing literature on relationships is the assumption that there is a dichotomy of relationships and that all relationships can be characterized as either short term or long term. Results of the current study show that not all human relationships fit within this dichotomy, as some relations (e.g., the booty-call relationship) incorporate characteristics of both short- and long-term relationships. Our results support previous suggestions that the booty-call relationship is a compromise relationship that benefits the sexes in different ways (e.g., Jonason et al., 2009 , 2011). However, the current study also extends previous research by establishing the necessity of a booty-calls mate’s physical attractiveness, kindness, and social level. Furthermore, the current study shows that both sexes considered the physical attractiveness of a booty-call mate a necessity, suggesting that both sexes could be using the booty-call relationship as a means of satisfying short-term sexual means (e.g., Jonason, 2013). Finally, although previous research has conceptualized the booty-call relationship as a compromise between men and women (e.g., Jonason et al., 2009), our findings indicate that perhaps it is only women, not men, who are doing the compromising.

## Appendix

### Physical Attraction

For the trait of physical attractiveness, there was a main effect of budget, *F*(1, 493) = 181.66, *p* = .001, $\eta_{\text{p}}^{2}$ = .27; gender, *F*(1, 493) = 33.65, *p* = .001; $\eta_{\text{p}}^{2}$ = .06; and relationship type, *F*(2, 493) = 49.28, *p* = .001, $\eta_{\text{p}}^{2}$ = .17. Post hoc analyses with the Bonferroni correction were conducted. For budget, post hoc analyses demonstrated people spent more mate dollars on physical attractiveness in the low budget condition (*M* = 32.12, *SE* = .72) compared to the high budget condition (*M* = 23.72, *SE* = .27), suggesting that this trait is considered a necessity, *p* = .001. For gender, men spent more on physical attractiveness (*M* = 30.51, *SE* = .77) than did women (*M* = 25.32, *SE* = .46), *p* = .001. In relation to relationship type, people spent more mate dollars on a booty-call mate’s physical attractiveness (*M* = 30.41, *SE* = .87) and a short-term mate’s physical attractiveness (*M* = 31.40, *SE* = .72) than they did on physical attractiveness in long-term relationships (*M* = 21.94, *SE* = .73), *p* = .001 and *p* = .001, respectively. In addition, there was no significant difference between the amount of mate dollars individuals spent on a booty-call and short-term mate’s physical attractiveness.

### Kindness

For the trait of kindness, there was a main effect of budget, *F*(1, 493) = 13.56, *p* = .001, $\eta_{\text{p}}^{2}$ = .03; gender, *F*(1, 493) = 38.37, *p* = .001, $\eta_{\text{p}}^{2}$ = .07; and relationship type, *F*(2, 493) = 27.43, *p* = .001, $\eta_{\text{p}}^{2}$ = .10. Post hoc analyses with the Bonferroni correction were conducted. For budget, post hoc analyses demonstrated that people spent more mate dollars on kindness in the low budget condition (*M* = 24.07, *SE* = .56) than they did in the high budget condition (*M* = 22.34, *SE* = .30), suggesting this trait is considered a necessity, *p* = .001. For gender, women spent more on kindness (*M* = 25.56, *SE* = .39) than did men (*M* = 20.84, *SE* = .65), *p* = .001. Finally, for relationship type, people spent more mate dollars on a long-term mate’s kindness (*M* = 26.48, *SE* = .62) than on a short-term mate’s kindness (*M* = 20.02, *SE* = .61), *p* = .001. In addition, people spent more mate dollars on a long-term mate’s kindness compared to a booty-call mate’s kindness (*M* = 23.12, *SE* = .74), *p* = .002. Finally, people spent significantly more mate dollars on a booty-call mate’s kindness than a short-term mate’s kindness, *p* = .004.

### Social Level

For social level, there was a significant main effect of budget, *F*(1, 493) = 93.16, *p* = .001, $\eta_{\text{p}}^{2}$ = .16; gender, *F*(1, 493) = 4.69, *p* = .031, $\eta_{\text{p}}^{2}$ = .01; and relationship term, *F*(2,493) = 6.24, *p* = .002, $\eta_{\text{p}}^{2}$ = .03. Post hoc comparisons with a Bonferroni adjustment were conducted. Post hoc results showed people spent more mate dollars on social level in the high budget condition (*M* = 18.59, *SE* = .31) compared to the low budget condition (*M* = 14.64, *SE* = .47), *p* = .001. This result demonstrates the characteristic of social level is considered a luxury. In addition, women spent more mate dollars on a mate’s social level (*M* = 17.36, *SE* = .35) compared to men (*M* = 15.87, *SE* = .59), *p* = .031. Finally, people spent significantly more mate dollars on a long-term mate’s social level (*M* = 18.06, *SE* = .56) than on a booty-call mate’s social level (*M* = 14.98, *SE* = .67), *p* = .001. No other comparisons were significant.
